# Multilevel Classification of Users’ Needs in Chinese Online Medical and Health Communities: Model Development and Evaluation Based on Graph Convolutional Network

**DOI:** 10.2196/42297

**Published:** 2023-04-20

**Authors:** Quan Cheng, Yingru Lin

**Affiliations:** 1 School of Economics and Management Fuzhou University Fuzhou China

**Keywords:** online medical health community, multilevel classification, graph convolutional network, cardiovascular disease, cardiovascular, China, online, medical, community, behavior

## Abstract

**Background:**

Online medical and health communities provide a platform for internet users to share experiences and ask questions about medical and health issues. However, there are problems in these communities, such as the low accuracy of the classification of users’ questions and the uneven health literacy of users, which affect the accuracy of user retrieval and the professionalism of the medical personnel answering the question. In this context, it is essential to study more effective classification methods of users’ information needs.

**Objective:**

Most online medical and health communities tend to provide only disease-type labels, which do not give a comprehensive summary of users’ needs. The study aims to construct a multilevel classification framework based on the graph convolutional network (GCN) model for users’ needs in online medical and health communities so that users can perform more targeted information retrieval.

**Methods:**

Using the Chinese online medical and health community “Qiuyi” as an example, we crawled questions posted by users in the “Cardiovascular Disease” section as the data source. First, the disease types involved in the problem data were segmented by manual coding to generate the first-level label. Second, the needs were identified by K-means clustering to generate the users’ information needs label as the second-level label. Finally, by constructing a GCN model, users’ questions were automatically classified, thus realizing the multilevel classification of users’ needs.

**Results:**

Based on the empirical research of questions posted by users in the “Cardiovascular Disease” section of Qiuyi, the hierarchical classification of users’ questions (data) was realized. The classification models designed in the study achieved accuracy, precision, recall, and F1-score of 0.6265, 0.6328, 0.5788, and 0.5912, respectively. Compared with the traditional machine learning method naïve Bayes and the deep learning method hierarchical text classification convolutional neural network, our classification model showed better performance. At the same time, we also performed a single-level classification experiment on users’ needs, which in comparison with the multilevel classification model exhibited a great improvement.

**Conclusions:**

A multilevel classification framework has been designed based on the GCN model. The results demonstrated that the method is effective in classifying users’ information needs in online medical and health communities. At the same time, users with different diseases have different directions for information needs, which plays an important role in providing diversified and targeted services to the online medical and health community. Our method is also applicable to other similar disease classifications.

## Introduction

The online medical and health community is the main platform for providing online consultation services, where users disseminate, obtain, use, and evaluate health [1-3]. Such a powerful interactive platform has a positive impact on patients’ daily disease control, self-health management, and emotional support [4-6]. However, the convenience of internet services and trust in online health information may affect users’ search behavior [7]. China’s internet user group is huge, and the level of medical knowledge of online medical users represented by middle-aged people is uneven. Groups with lower health literacy will face higher risks [8]. By observing the way users ask questions in many online medical and health communities, we found that they often ask questions by independently selecting departments and disease types. The accuracy of their choices is biased, which not only affects medical professionals in answering questions but also may lead to a variety of data in which other patients appear when collecting information. These drawbacks will further reduce users’ trust in online health information and increase the risk of information acquisition. Users are the main body of the online medical and health community, and whether the community can accurately understand the health needs of users is the key to providing users with accurate health information [9].

Recent studies on the needs of users in online medical and health communities mainly focused on the classification of information needs. Empirical studies are generally conducted on specific diseases (eg, chronic diseases such as osteoarthritis [10], diabetes mellitus [9], cardiovascular disease [11]) and certain diseases with high mortality rates (eg, breast cancer [12,13] and lung cancer [9]). The main methods for data collection are questionnaire survey [14] and machine learning–based methods [11,12,15-17]. Questionnaire survey is used to assess patients’ information needs through interviews and other means [13,18], which often has the problem of unbalanced results due to small and uneven research samples. Therefore, machine learning–based methods such as text mining and topic recognition have gradually become the most common methods to study users’ information needs. Qian and Gui [19] applied word frequency analysis to identify the information needs of users in the elderly health community, which is mainly divided into 4 themes: coping with aging, diet and nutrition, physical exercise, and mental health. Based on the coword analysis and the latent Dirichlet allocation topic model, Wang et al [15] found that during the COVID-19 pandemic, Chinese online medical and health community users had the highest demand for information about disease symptoms [15]. Pérez-Pérez et al [16] explored the most frequently mentioned symptoms of the disease and the distribution of patient emotions through semantic analysis of patients’ discussions about the disease in the Twitter intestinal disease community [16]. Luo et al [11] realized automatic topic identification of user requirements through a text clustering algorithm. To identify the topic of information needs, McRoy et al [12] applied data coding and random forest–based methods for supervised text classification of data postings by breast cancer survivors to assess their unmet information needs from their own perspectives and to identify gaps between information needs and current education materials. Guo et al [20] established a topic-based classification model using manual annotation to classify users’ information needs into 6 categories and 4 levels and used machine learning to achieve automatic classification.

However, all the aforesaid studies only focused on identifying the types of users’ information needs, ignoring the importance of text classification from the perspective of disease. Chen [17] used clustering algorithms to analyze communities with 3 diseases (namely, breast cancer, diabetes mellitus, and fibromyalgia) and explored the changing nature of patients’ information needs in different communities as the disease develops. Hong et al [9] used a content analysis approach to study questions from patients with diabetes and hepatitis. It was found that the main concerns of patients with diabetes were related to treatment, whereas the more frequently asked questions by patients with hepatitis focused on diagnosis [9].

With the development of artificial intelligence, deep neural networks have been widely used in various natural language processing tasks. Graph convolutional network (GCN) is a generalization of convolutional operations from structured mesh data to unstructured graph data [21], which has been extensively studied in many fields, such as traffic network prediction [22], simulation of multidrug side effects [23], and personnel reidentification [24]. In recent years, it has also been applied in text classification [25,26], and its effectiveness in Chinese text classification has been proved by some studies [27]. Yao et al [28] constructed text graphs based on word co-occurrence, using documents and words as nodes and GCN for semisupervised text classification, and obtained more advanced classification results in most types of texts. However, Gao and Huang [29] considered text contextual information and improved the general GCN text classification by combining graph embedding with bidirectional encoder representations from transformers (BERT) embedding using GCN with a gating mechanism to achieve contextual encoding acquisition [29]. To solve the problems of sparse and insufficiently marked data in short texts, Zhao et al [30] proposed a multihead pooling-based GCN for semisupervised short textbook classification, focusing on the structural information and important nodes of the graph to achieve powerful classification performance with low computational cost [30]. Ye et al [21] applied the GCN model to short-text classification by inputting both word and document nodes trained by the GCN into bidirectional long short-term memory or other classification models for further classification of short textbooks.

To solve the aforesaid problems, this paper constructed a multilevel classification system for users’ needs from multiple perspectives. Taking users’ question(s) on China’s online medical and health community as the data source, the semiautomated method of “manual coding + text clustering” was used to extract the first-level disease label and the second-level information demand label. The GCN model was used to classify users’ data and evaluate the model proposed. In this way, users’ data can be observed from both subjective and objective perspectives so that medical professionals can be more targeted when answering questions, while other users can quickly identify the required information. In turn, the quality of online medical and health information is improved so that the online medical and health community can better provide medical and health information services [31].

The major contributions of this paper are summarized as follows:

- This study proposes a multilevel classification method. At present, research tends to target the needs of users in a certain aspect, such as emotional needs or information needs. Different physical conditions and treatment plans caused by different diseases lead to different information needs of different users. Through the multilevel classification structure of this paper, disease-type classification and information demand classification were performed so that users can conduct more targeted information retrieval.
- This study conducted semiautomated label identification. We used a manually coded approach to segment user diseases. Combined with other related research methods, K-means text clustering was used to generate different theme text sets to identify users’ information needs. This method of labeling reduces the subjective influence of manual coding and improves the speed of label recognition.

The rest of this paper is organized as follows: The “Methods” section introduces the multilevel classification system and the implementation of a multilevel classification algorithm for users’ requirements. In the “Results” section, the experimental results are analyzed in-depth. Finally, we discuss the conclusions of the study and the suggestions for future development.

## Methods

### Overview

This section introduces the process of constructing a multilevel classification system for users’ needs and the implementation of a multilevel classification algorithm for users’ needs.

### Data Collection

Our data were collected from “Qiuyi” [32]. Founded in 2010, Qiuyi is a medical website that combines practical tools (eg, online registration, appointments, drug inquiry) and personalized medical services and suggestions (eg, disease encyclopedia, emotional communication, answering questions) in China. As of 2019, cardiovascular disease was the leading cause of death among urban and rural residents, and the burden of this disease was on the rise. Therefore, we utilized users’ questions (a total of 25,999 data points) in the “Cardiovascular Disease” section from November 3, 2011, to December 28, 2020, as the data source for this study. For each question, the data contain fields such as the title of the user’s question, question time, detailed description of the question, treatment received, and the help the user hoped to obtain.

This article used Python’s Beautiful Soup library (Python Software Foundation) for web page crawling, parsing useful information about the page, and storing it in a database. To ensure the anonymity of users’ question, the crawled content of our work is a public question that can be viewed without logging in to the website.

Table 1 shows the distribution of data on users’ questions about cardiovascular diseases from 2011 to 2020. It can be seen that the users’ data continued to grow from 2011 to 2015, and the reason for this was the gradual increase in internet users; until 2016, with the increase in a large number of online medical and health communities, there was a sharp decrease in the number of questions posted due to user diversion.

**Table 1:** Statistics of users’ questions in the “Cardiovascular Disease” section of Qiuyi from 2011 to 2020 (N=25,999).

Year	Count, n
2011	31
2012	621
2013	935
2014	948
2015	8539
2016	61
2017	403
2018	337
2019	7195
2020	6929

### Ethics Approval

In line with the regional requirements [33], the study did not require ethical approval because the data extracted from the users’ questions are public content, freely available on the internet, and the study was conducted using anonymized information data. However, even without the need to provide an ethical statement, we still complied with the relevant privacy and data protection legislation to ensure the security and legality of data. In our view, these measures are sufficient to protect the privacy of individuals, while avoiding the abuse or invasion of privacy caused by the failure to provide an ethical statement.

### Preprocessing

From the 25,999 crawled data, we randomly selected 10,000 data points as the experimental data and relied on Python’s Jieba thesaurus for accurate pattern word separation. We downloaded the thesaurus of topics related to medical terms and customized dictionaries for word segmentation to preserve proper nouns in the data. We then combined the stop thesaurus for deactivated word processing, filtering out numbers, punctuation marks, mood words, among other purposes.

Feature extraction is performed by calculating the term frequency-inverse document frequency (TF-IDF) values of words. TF-IDF is a common weighting technique used in information retrieval and data mining. In this article, it was primarily used to assess the importance of words in a user’s question data set. The importance of a word increases with the number of times it is in the document, but at the same time decreases with the number of times it is in the corpus. TF indicates the frequency with which a word appears in all documents. IDF can be obtained by dividing the total number of files by the number of files containing the word and then taking the logarithm base 10 of the resulting quotient.

### Multilevel Classification System Construction

#### Annotation Process

The “Cardiovascular Diseases” section involves many types of diseases, such as heart disease, vascular diseases. The subdivision of disease types helps to enhance the relevance of information. The Chinese National Standard “GB_T14396-2016 Classification Codes of Diseases” [34] and MeSH (medical subject headings) were combined as annotation rules to manually annotate the disease categories of the data set. The MeSH, an authoritative subject heading list compiled by the US National Library of Medicine, provides a hierarchy of cardiovascular disease names in the subject heading list. To reduce the labeling dimension, disease types in the second level of cardiovascular disease were identified in this paper. Diseases such as “congenital heart disease” and “vascular malformations” were classified as “cardiovascular abnormalities.” “Arrhythmia,” “cardiomyopathy,” “endocarditis,” and other diseases were classified as “heart diseases,” while “hypertension,” “cerebrovascular diseases,” etc were classified as “vascular diseases.” Other data that only describe the symptoms and do not include the name of the disease, those related to surgery or medication, and those not related to cardiovascular diseases were classified as “other cardiovascular diseases.”

#### Clustering and Topic Identification

Automatic identification of second-level labels for user requirements is performed by text clustering. The main text clustering methods that have been proposed thus far are K-means clustering, hierarchical clustering methods, latent Dirichlet allocation topic clustering models, and planar division methods. In this study, K-means clustering was selected as the main method for label identification at the second level of users’ needs. The K-means algorithm is an unsupervised iterative clustering algorithm. Taking document clustering as an example, the implementation idea of this algorithm is as follows:

1. Set the number of clusters to *k*. From the document set of {*D*_1_, *D*_2_, *D*_3_,…, *D_n_*}, randomly select *k* as the initial clustering center {*A*_1_, *A*_2_, *A*_3_,…, *A_k_*};
2. Calculate the distance between the remaining documents in the document set *D* and the cluster center {*A*_1_, *A*_2_, *A*_3_,…, *A_k_*} separately and divide each document into the cluster with the smallest distance from it;
3. Repeat step 1 until the document in the document set *D* is divided into *k* text clusters;
4. Recalculate the centroids of each text cluster and update the cluster centers {*A′*_1_, *A′*_2_, *A′*_3_,…, *A′_k_*};
5. Repeat steps 2-4 until convergence and output cluster division *C*={*C*_1_, *C*_2_, *C*_3_,…,*C_k_*}.

### Classification Algorithm

The GCN model is chosen as the classification model. GCN is a method of using convolutional computation on graph data and is a generalization of convolutional neural networks (CNNs) to the graph domain, which generates new node representations by aggregating node information using information from edges in the graph. For graphs *G*=(*V*, *E*), where *V* denotes the set of nodes and *E* represents the set of edges, the formula is given as:

*L*^(^*^j^*^+1)^ = *ρ*[*ÃL*^(^*^j^*^)^*W_j_*] **(1)**

Further, we consider the following:

- *A* is the graph of the *n*-order adjacency matrix, and if edges exist at the nodes *v_i_*, *v_j_*, then *A_ij_*=1. To update the features of the node itself, it is necessary to introduce self-join, that is, a node to the node itself exists on the edge, *A*=*A*+*I*;
- *Ã* is the normalized adjacency matrix. Normalizing the adjacency matrix eliminates the problem of large differences in features after aggregation due to differences in the number of neighboring nodes. *Ã*=*D*^(–1/2)^*AD*^(–1/2)^; here *D* is the degree matrix of the graph;
- *X* ∈ *R*^*n*×*m*^ is a matrix containing *n* nodes and their initial eigenvectors, with *m* being the dimensionality of the eigenvectors and *L*^0^=*X*;
- *W* ∈ *R*^*m*×*k*^ is the weight matrix, with *W*_j_ being the weight matrix of the *j* graph convolution layer; and
- *ρ* is the activation function, commonly known as the sigmoid, tanh, or rectified linear unit (ReLu) function.

TextGCN is an application of a graph neural network in text classification that involves constructing a heterogeneous graph for all texts, which contains all document nodes and word nodes, and the labels of document nodes can be passed to other nodes through neighboring nodes.

### Constructing Heterogeneous Images

After data preprocessing, the advantage of the data obtained is leveraged to build a large heterogeneous map that includes document nodes and word nodes. The weight between the document node and the word node *A_ij_* is equal to the TF-IDF value. Pointwise mutual information (PMI) values are used between word nodes and document nodes to calculate *A_ij_*=PMI. These values can define the mutual information of weight reuse points of edges between 2 word nodes.


PMI_(_*_i_*_,_*_j_*_)_ = log [*p*(*i*,*j*)/*p*(*i*)*p*(*j*)] **(2)**

*p*(*i*,*j*) = [#*W*(*i*,*j*)/#*W*] **(3)**

*p*(*i*) = [#*W*(*i*)/#*W*) **(4)**

To utilize global co-occurrence information, a sliding window of fixed size is used for all documents. #*W* denotes the total number of sliding windows in the corpus, #*W*(*i*) denotes the number of sliding windows containing the word *i*, and #*W*(*i*,*j*) denotes the number of sliding windows containing both words *i* and *j*. PMI(*i*,*j*)>0 indicates a high semantic relevance between words and conversely a low or no semantic correlation. Therefore, when constructing the heterogeneous graph, only edges between the word nodes in which PMI(*i*,*j*)>0 are added and the sliding window size #*W* is set to 20. The sparse matrix was saved as a graph, and a heterogeneous graph with 25,590 nodes and 1,806,090 edges was obtained.

### Building a GCN Model

In this paper, we constructed a 2-layer GCN model, fed the constructed heterogeneous graph into the model, and used the softmax() method for classification. The embedding size of the first convolutional layer was set to 300, and ReLu was used as the activation function, thereby increasing the nonlinearity of the model and strengthening the learning ability of the network. The dropout parameter is set during training to temporarily drop the neural network units from the network according to a certain probability, thus improving the model generalization ability and preventing overfitting. The output of the first-layer label prediction is spliced with the input layer of the second-layer label prediction, and finally, the output of both layers of labels is returned simultaneously, thus realizing multilayer classification.

### Evaluation Metrics

In general, the performance evaluation metrics for classifiers include both efficiency and effectiveness. Efficiency refers to the time used by the classifier during the process of training the model and the time needed to make predictions on the test data, while there are various metrics to evaluate the effectiveness. In this paper, from the perspective of effect, the results of multilevel classification are evaluated, mainly including accuracy, *F*_1_-score, precision, and recall. The evaluation dimensions were (1) evaluation of the effectiveness between the GCN and combined classifiers and (2) effectiveness assessment between multilevel classification and single-level classification.

Accuracy measures the percentage of the total quantity that is correctly predicted. The more quantities that are correctly predicted, the higher the accuracy.


Accuracy = (TP + TN)/(TP + TN + FP + FN) **(5)**

where TP is the number of true cases (predicted positive and actual positive); TN is the number of true negative cases (predicted negative and actual negative); FP is the number of false-positive cases (predicted positive and actual negative); and FN is the number of false-negative cases (predicted negative and actual positive).

Recall is defined as the proportion of positive cases (TP) correctly determined by the model to all positive (TP + FN) cases in the data set.


Recall = TP/(TP + FN) **(6)**

Precision is the percentage of true cases (TP) among all positive cases (TP + FP) judged by the model.


Precision = TP/(TP + FP) **(7)**

The *F*_1_-score is the sum of precision and recall, and recall is considered to be as important as precision. In this paper, we used the macroaverage algorithm to calculate the *F*_1_-score.

## Results

### Multilevel Classification System for Users’ Information Needs

In the first level of labels obtained by manual annotation, 26.64% (2664/10,000) were identified as asking questions related to other cardiovascular diseases, 16.45% (1645/10,000) were identified as asking questions about cardiovascular abnormalities, 28.24% (2824/10,000) were identified as asking questions related to heart disease, and 28.67% (2867/10,000) were identified as asking questions related to vascular disease.

Table 2 presents the keywords and sample data for each category of users’ needs, after applying K-means for text clustering, in the second level of the cardiovascular disease section. The 8 topics of users’ information needs were “Disease causes” (588/10,000, 5.88%), “Self-reported symptoms” (15.89%, 1589/10,000), “Clinical signs and symptoms” (943/10,000, 9.43%), “Clinical examination” (1041/10,000, 10.41%), “Prevention and recuperation” (1928/10,000, 19.28%), “Operative treatment” (1188/10,000, 11.88%), “Drug treatment” (1351/10,000, 13.51%), and “Therapeutic method” (1372/10,000, 13.72%). The top 5 terms with TF-IDF values were used as keywords for the information demand category. Users’ information demand for cardiovascular disease mainly tends to be in the areas of “Daily prevention and maintenance” and “Patient symptom self-report.” This may be because cardiovascular disease is a chronic disease, and people are more focused on the impact of their behavioral habits in daily life on the disease.

**Figure 1:**
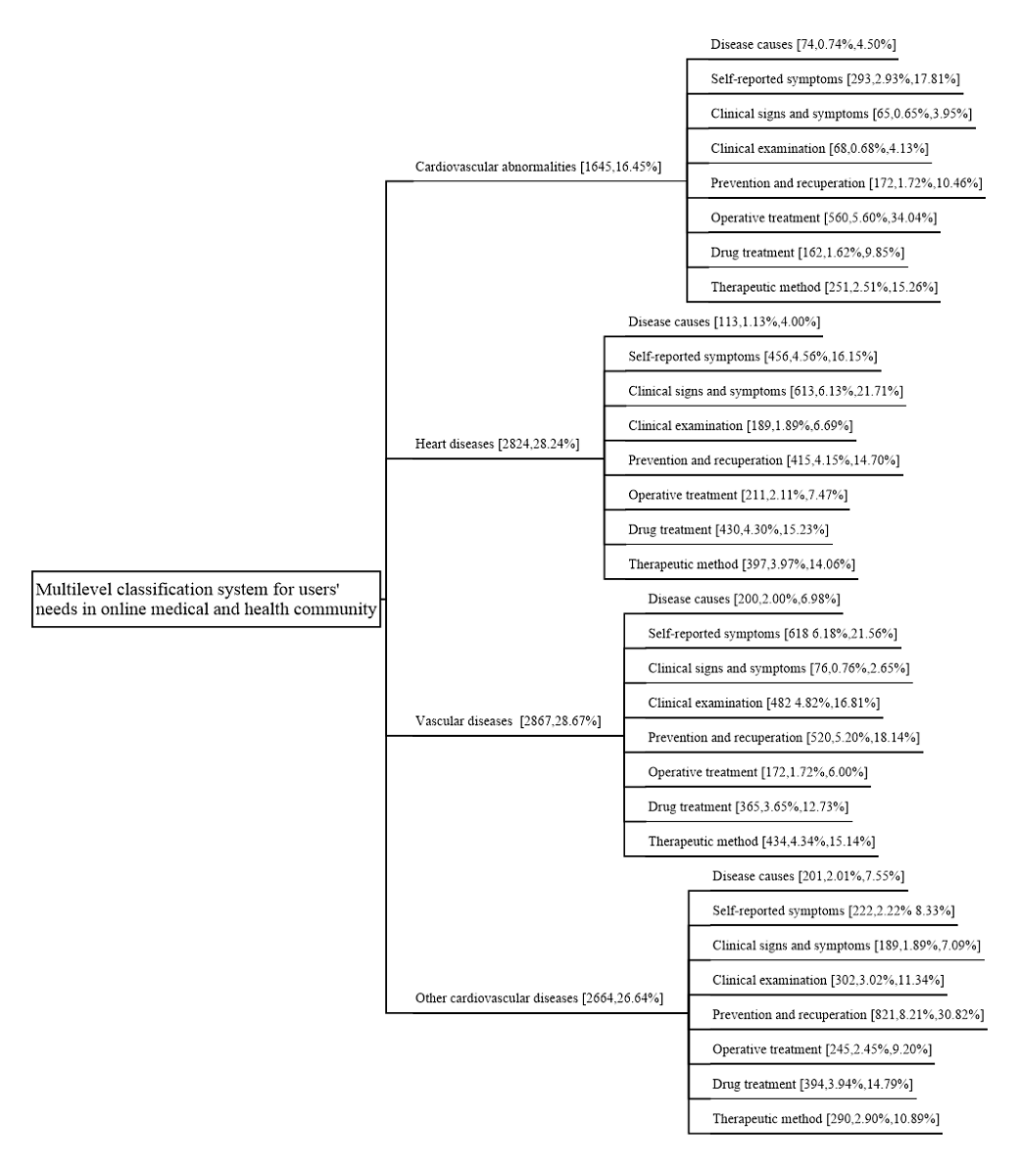
Multilevel classification system for users' needs in online medical and health community. The 3 sets of values in parentheses represent the number of data bars for the topic, the proportion of the total number of data bars, and the proportion of the number of data bars in the parent node (N=10,000).

The final multilevel classification system of users’ needs for the online medical and health community is shown in Figure 1, with 4 types of disease topics in level 1 and 8 types of requirement topics in level 2. Vascular diseases mainly include hypertension and arterial occlusive diseases, and users are more interested in the information on “Daily prevention and maintenance.” Cardiovascular abnormalities mainly include congenital heart disease, and users are more interested in the information on “Clinical examination.” It can be seen that different users have different information needs for different diseases, so this issue should be considered in practical applications to differentiate the information service provision for different diseases. For users who want accurate and fast answers, knowing more granular, specific needs can help the site assign more experienced medical professionals accordingly.

**Table 2:** Category keywords and examples of users’ information needs in the “Cardiovascular Disease” section of Qiuyi (N=10,000).

Category	Meaning	Keywords	Sample excerpts
Operative treatment	Users inquire about surgical procedures, postoperative recovery, surgical costs, success rates, etc	Surgery, careful examination, treatment, causes, surgery	*What to look for after valve replacement surgery for valvular disease*
Drug treatment	Users ask questions about medications for diseases or side effects of medications	Examinations, medications, electrocardiogram, routine, treatment	*What are the side effects of bei jing ling hao and how to relieve them?*
Self-reported symptoms	Users consult whether they are ill by describing their physical condition and symptoms	Feelings, body, discomfort, causes, dizziness	*What’s wrong with a red face and dizziness? The heart is weak, the blood pressure is slightly higher, the high pressure is about 150 (the patient is 64 years old), there are symptoms of panic and dizziness, and the face is often red with headaches.*
Clinical examination	Users can have consultations regarding their condition by describing about their clinical examination results or inquiring what tests are required by the disease description	Body, health check, high, impact, danger	*Classmates were found to have sinus irregularities. After careful examination, the teacher took the electrocardiogram and said that the PR interval prolongation was diagnosed as sinus arrhythmia. Hello, will the sinus arrhythmia PR interval prolong?*
Therapeutic method	Users consult treatments such as surgery, drugs, or folk remedies or the effectiveness of a certain treatment	Treatment, condition, effect, method, time	*What should I do to treat my hypertensive nephropathy? I have hypertensive nephropathy, how should I implement the treatment to save my life?*
Prevention and recuperation	Users consult on how to prevent the occurrence of diseases or inhibit the development of diseases, such as eating habits, daily routines	Attention, diet, food, usual, spicy	*I just have low blood pressure now and then I want to do a diet regimen, How to do low blood pressure diet conditioning?*
Disease causes	Users consult about the cause or type of disease	What’s going on, take medication, feel, uncomfortable, effect	*What is the cause of swollen legs and feet in hemiplegia?*
Clinical signs and symptoms	Self-diagnosis of users by consulting clinical symptoms of certain disease	Symptoms, breathing, presentation, findings, sensations	*I am male and 46 years old. In the first half of the year found sweating, dry heat feeling, and shortness of breath. What are the possible symptoms of coronary heart disease?*

### Empirical Research of Multilevel Classification of Users’ Needs Based on GCN

We selected 6 sets of parameter combinations commonly used in studies for training and comparing the experimental results. During the training process, we trained Text-GCN for a maximum of 200 epochs and introduced an early stopping mechanism to monitor the validation loss. The training process is stopped if the validation loss does not decrease for more than 10 consecutive cycles. Through the comparison experiment, it can be seen that the final set of dropout=0.3 and learning rate=0.01 gives the best classification results. The model classification effect obtained under different hyperparameters is shown in Table 3. The multilevel GCN model was trained according to the optimal hyperparameters, and the results of the multilevel classification of users’ needs obtained by the experiment are shown in Table 4. Aiming at the lowest level of labels required by users, the model proposed in this study achieved accuracy, accuracy, recall, and *F*_1_-score of 0.6265, 0.6328, 0.5788, and 0.5912, respectively.

**Figure 2:**
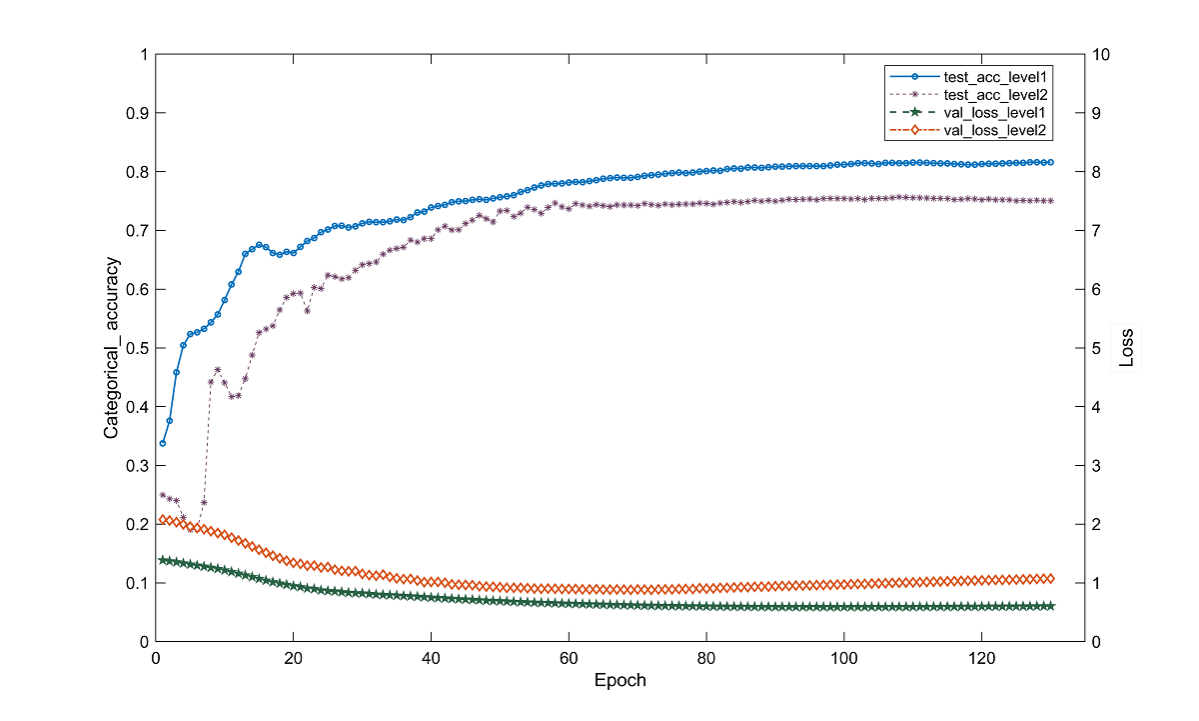
The learning curve of the multilevel GCN. GCN: graph convolutional network; test_acc_level1: level 1 accuracy; test_acc_level2: level 2 accuracy; val_loss_level1: level 1 loss value; val_loss_level2: level 2 loss value.

Figure 2 shows the learning curve of the multilevel GCN. It can be seen that after 100 iterations (epoch=100), the model tends to be stable, the loss function at the second level of the test set has an upward trend in the later stage, and there is slight overfitting.

**Table 3:** Hyperparameters of graph convolutional network and corresponding evaluation indices.^a^

Parameters	Combination					
	1. Learning rate	2. Dropout	3. Accuracy	4. Precision	5. Recall	6. *F*_1_-score
1. Learning rate	0.05	0.01	0.005	0.05	*0.01*	0.005
2. Dropout	0.5	0.5	0.5	0.3	*0.3*	0.3
3. Accuracy	0.6085	0.6220	0.6145	0.5965	*0.6265*	0.6130
4. Precision	0.6057	0.6283	0.6213	0.5672	*0.6328*	0.6186
5. Recall	0.5696	0.5711	0.5514	0.5278	*0.5788*	0.5465
6. *F*_1_-score	0.5769	0.5847	0.5640	0.5306	*0.5912*	0.5601

^a^The italicized values indicate that this parameter combination obtained the optimal model effect, which will be used for model training in subsequent demonstrations.

**Table 4:** Demonstration of multilevel classification effect based on graph convolutional network.

Level	Accuracy	Precision	Recall	*F*_1_-score
Level_1^a^	0.8215	0.8323	0.8264	0.8288
Level_2^b^	0.7535	0.7613	0.7538	0.7566
Final_level^c^	0.6265	0.6328	0.5788	0.5912

^a^Indicates the prediction of the first-level label.

^b^Indicates the prediction of the second-level label.

^c^Final_level indicates the final prediction result combining the first- and second-level labels.

### Comparison Verification

To verify the effectiveness of the GCN in multilevel classification, the bottom prediction result (Final_level) is taken for multilevel comparison validation of different classification models and single-level comparison validation of the same classification model.

### Multilevel Classification Comparison Validation

To verify the effectiveness of the multilevel GCN classification model used in this paper, 2 hierarchical classification models were utilized for multilevel classification and testing in the case of the same data set. One is to construct a homogeneous combined classifier using naïve Bayes (NB) as the base classifier, and the other is to use a multilevel CNN model named hierarchical text classification CNN (HCCNN). As shown in Table 5, a consistent classification effect evaluation metric was used to compare the bottom label prediction effect.

**Figure 3:**
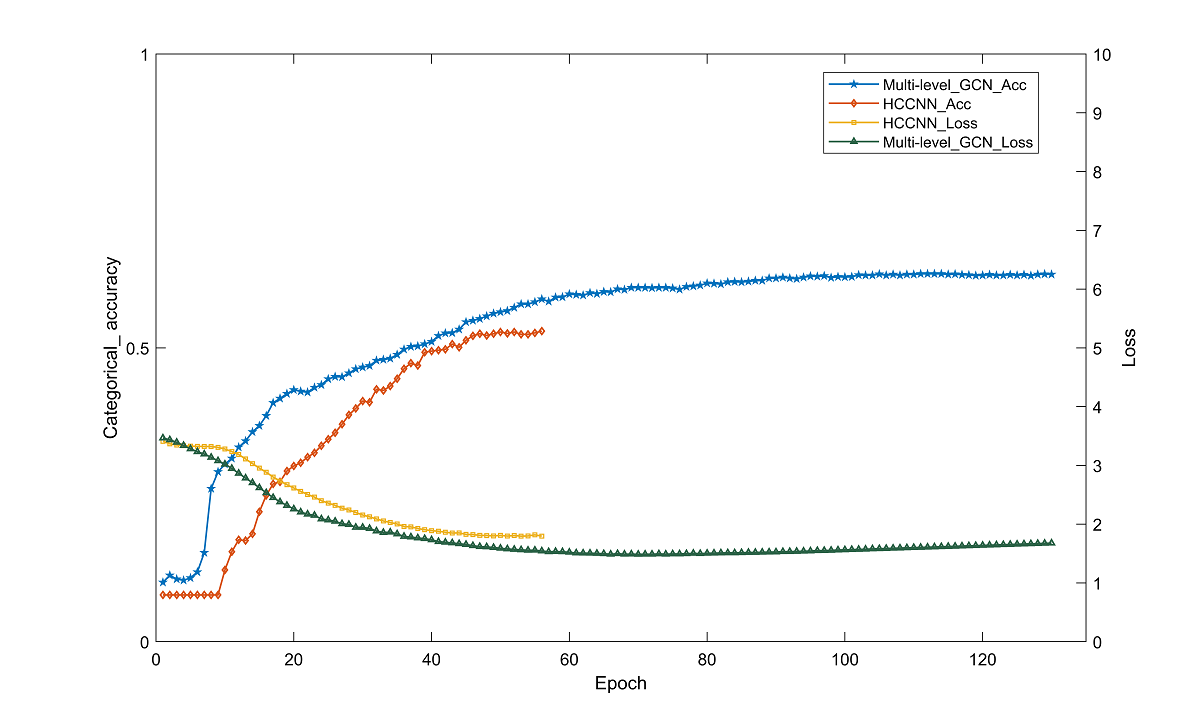
Two hierarchical classification models: loss function and accuracy curve. CNN: convolutional neural network; GCN: graph convolutional network; HCCNN_Acc: hierarchical text classification CNN model accuracy; HCCNN_Loss: hierarchical text classification CNN model loss value; Multi-level_GCN_Acc: accuracy of the multilevel graph convolutional network model; Multi-level_GCN_Loss: loss value of the multilevel graph convolutional network model.

According to the comparison results in Table 5, it can be seen that the results of the training and testing of the multilevel GCN model used in this paper are better than those of the NB hierarchical classifier and the HCCNN, with accuracy improvements of 2.4% and 9.8%, respectively. Figure 3 shows the accuracy and loss value change curves of the final label prediction of the multilevel GCN and HCCNN models. The figure shows that the HCCNN model is forced to stop and reach the optimum at the 56th round of iteration, and the model accuracy is more different from that of the multilevel GCN.

**Table 5:** Demonstration of each multilevel classification model.^a^

Models	Accuracy	Precision	Recall	*F*_1_-score
TF-IDF^b^ + NB^c^	0.6025	0.6361	0.4853	0.4994
Word2vec + HCCNN^d^	0.5285	0.3817	0.4966	0.3840
Multilevel GCN^e^	*0.6265*	*0.6328*	*0.5788*	*0.5912*

^a^Best results (proposed model) are italicized.

^b^TF-IDF: term frequency-inverse document frequency.

^c^NB: naïve Bayes.

^d^HCCNN: hierarchical text classification convolutional neural network.

^e^GCN: graph convolutional network.

### Single-Level Classification Comparison Validation

To verify whether there is an advantage of multilevel classification over the single-level classification of the bottom labels, the multilevel GCN classification results were compared with the single-level GCN classification results. The 2 layers of labels are combined to generate the lowest labels in a total of 32 categories. Table 6 shows the performance evaluation and classification results obtained.

**Figure 4:**
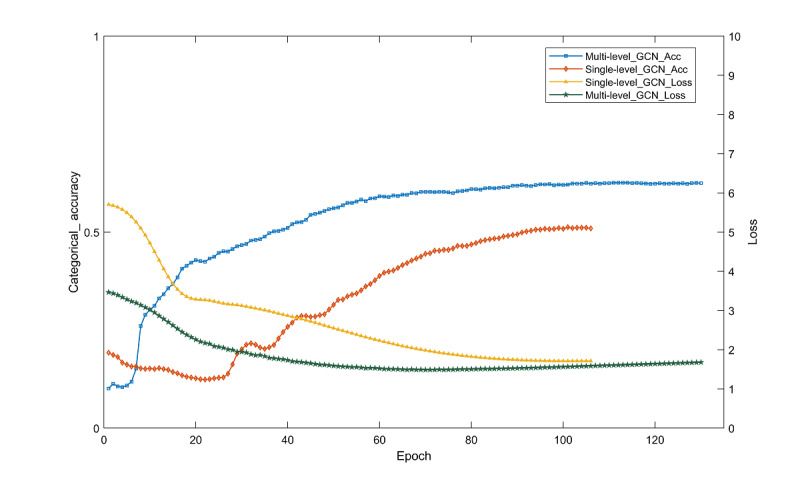
Single-level and multi-level model loss function and accuracy change curve. GCN: graph convolutional network; Multi-level_GCN_Acc: accuracy of the multilevel graph convolutional network model; Multi-level_GCN_Loss: loss value of the multilevel graph convolutional network model; Single-level_GCN_Acc: accuracy of the single-level graph convolutional network model; Single-level_GCN_Loss: loss value of the single-level graph convolutional network model.

As seen from Table 6, the multilevel GCN model showed an increase in accuracy, precision, recall, and *F*_1_-score of 11.4%, 14.02%, 14.94%, and 14.94% compared with the single-level GCN model. As shown in Figure 4, the loss function and accuracy change curves of the final label prediction for the single-level GCN model show that the single-level GCN model forces the end of the iteration at the 106th time. In the case of using the same classification method, the classification results obtained by performing multilevel classification are better.

**Table 6:** Demonstration of single-level and multilabel classification.^a^

Model	Accuracy	Precision	Recall	*F*_1_-score
Single-level GCN^b^	0.5125	0.4926	0.4294	0.4418
Multilevel GCN	*0.6265*	*0.6328*	*0.5788*	*0.5912*

^a^Italicized entries are the optimal results of the comparison experiment.

^b^GCN: graph convolutional network.

## Discussion

### Principal Findings

In the era of big data and artificial intelligence, internet health and medical treatment have become a necessary supplement to human’s traditional health management and disease diagnosis and treatment model, but there are also a series of major challenges. Among them, the rapid classification of users’ needs information and the efficient matching of expert diagnosis and treatment and health guidance information are important to realize online health guidance and diagnosis and treatment. In this study, a semiautomated label recognition method based on “text clustering + manual recognition” was proposed to explore the multidimensional health needs classification system of users in an online medical and health community. At the same time, a double-layer GCN classification model was constructed to meet the needs of various users in the “Cardiovascular Disease” section of the online medical and health community. The first level covered 4 disease types, while the second level covered 8 health information demand types. To the best of our knowledge, this study is the first to propose a classification system that combines disease needs and medical information needs. By analyzing users’ information needs, we found that patients with various diseases have obvious differences in information needs. For example, users with vascular diseases are more interested in the information about “Daily prevention and care,” whereas users with cardiovascular abnormalities are more interested in the “Clinical examination” message. In addition, according to a previous study [35], users of the “Tumor” section of Qiuyi pay more attention to the information in the direction of “treatment,” whereas users of the “Cardiovascular Disease” section in this study paid more attention to the information in the direction of “daily prevention and nursing.” As a result, there is an increased need for differentiated information services in online medical and health communities.

The GCN model constructed in this study was used for multilevel classification, and the data set of Qiuyi, an online medical and health community in China, was used for empirical research. In the empirical research, the traditional machine learning algorithm NB and the hierarchical text classification model HCCNN were introduced to perform the multilevel classification comparison experiment. Using TF-IDF to extract word vectors, NB was introduced to construct the model of combinatorial classifier, with accuracy, precision, recall, and *F*_1_-score of 0.6025, 0.6361, 0.4853, and 0.4994, respectively. The accuracy, precision, recall, and *F*_1_-score of 0.5285, 0.3817, 0.4966 and 0.3840 were obtained by combining Word2Vec with HCCNN. However, the multilevel classification model of GCN constructed in this study achieved 0.6265, 0.6328, 0.5788, and 0.5912 in the indices of accuracy, precision, recall, and *F*_1_-score. Compared with the TF-IDF + NB method, it presents a small improvement in accuracy and precision, but a large improvement in recall and *F*_1_-score. Compared with Word2Vec + HCCNN, the GCN model presents obvious advantages. The experimental results show that the model based on GCN performs better than other models in the multilevel classification of the data set in this paper. Second, to verify the effectiveness of multilevel classification, the 2 layers of labels required by users are fused to conduct a single-level classification. The multilevel classification presented more advantages compared with the single-level classification in the indices of accuracy (11% advantage), precision (14% advantage), recall (14% advantage), and *F*_1_-score (15% advantage), which indicates the necessity of multilayer classification. In general, the model constructed in this study shows good performance in classifying users’ needs in online medical and health communities. The advantage of this study is that it is the first classification model that includes both disease types and medical information needs, and it is based on an online medical and health community containing a large amount of information, which ensures the universality of the study.

### Limitations and Future Research

This article also has some limitations that must be considered. First, in terms of how to improve the adaptability and robustness of the multilevel classification model, this study has the possibility of further improvement. In this study, only the questions asked by users in the “Cardiovascular Disease” section of the online medical and health community were selected as the object for analysis. The sample type is single, the number of samples is limited, and the architecture of health information requirements is relatively simple, resulting in bottlenecks in the adaptability and robustness of the multilevel health needs classification algorithm and framework. For multiple disease types and large sample data, the efficiency and effectiveness of the hierarchical classification model proposed in this study need to be further verified.

Second, in terms of sample data selection, this study focuses on the data (question) of a single disease plate on a single platform, without considering the data fusion among multiple plates across platforms and disease types. While analyzing the information about users’ demand for questions, we only focused on the identification of a single disease type, which is a means to artificially reduce the complexity of natural language processing, at the cost of all the semantic features in users’ health questions. For example, by observing the data, it can be found that some users asked about information that does not belong to the category of cardiovascular diseases at all, whereas the data extracted in this paper only relate to the category of cardiovascular diseases. Some questions involved both cardiovascular and tumor plates, but the latter could not be identified during classification. In subsequent studies, the data volume can be increased to obtain a larger scale of empirical studies. The fusion data objects across disease types can be considered to conduct classification studies with a higher degree of complexity.

Third, this study has the possibility of further upgrading the classification level while constructing the disease classification system. In this study, for the category of cardiovascular diseases, we established a 2-level manual labeling and category system according to the Chinese national standard “GB_T14396-2016 Classification Codes of Diseases” and MeSH. Therefore, the multilevel classification model based on GCN constructed in this study is only applicable to the classification problem of a 2-layer category system. Thus, we should aim to build a health information classification model that satisfies more levels. This will enable users’ needs at a lower level of granularity to be mined to achieve the refinement and precision of users’ demand identification. Finally, the experimental process of this study also has some areas that can be further improved. In the future, we can study some more appropriate clustering methods and compare their performance applicable to our data set, so as to further improve the accuracy of classification methods and try more classification methods for comparative analysis.

### Conclusions

This study focused on the multilevel classification of users’ needs in an online medical and health community and constructed a multilevel classification system of users’ needs using a semiautomatic label recognition method. When applied to the data extracted from Qiuyi, an online medical and health community in China, the performance of the multilevel GCN classification model constructed was superior to other models, which can better learn the category features of the text asked by users and thus make a more accurate prediction. Compared with the existing research methods, the classification system we constructed not only more accurately locates the needs of users but also provides the direction for users’ information collection, which also helps medical professionals to provide higher quality information. To sum up, this study provides certain solutions for improving users’ experience and other types of multilevel classification tasks.

In the future, the multilayer classification framework of this study will be evaluated in more online medical and health communities and disease sections, and more advanced algorithms will be considered to further improve the performance of the classification method.

## Abbreviations

BERT: bidirectional encoder representations from transformers
CNN: convolutional neural network
GCN: graph convolutional network
HCCNN: hierarchical text classification convolutional neural network
MeSH: medical subject headings
NB: naïve Bayes
TF-IDF: inverse document frequency
PMI: pointwise mutual information
ReLu: rectified linear unit

## Data Availability

The data are available at [36].
